# Estimating injury incidence and describing self-rated physical and mental health during 12 weeks of walking football for older (60+) men and women with low physical activity levels

**DOI:** 10.1186/s13102-026-02224-4

**Published:** 2026-10-30

**Authors:** Andreas Caspers, Helena Andersson, Manne Godhe, Dan Fransson, Mats Börjesson, Elin Ekblom-Bak

**Affiliations:** 1 Department of Food and Nutrition, and Sport Science, University of Gothenburg, Box 300, Gothenburg, 405 30 Sweden; 2 Department of Community Medicine and Rehabilitation, Section of Sports Medicine, Umeå University, Umeå, Sweden; 3 Department of Physical Activity and Health, The Swedish School of Sport and Health Sciences, Stockholm, 114 33 Sweden; 4 Department of Molecular and Clinical Medicine, University of Gothenburg Sahlgrenska Academy, Gothenburg, Sweden; 5 Department of Medicine, Geriatrics and Acute Medicine, Sahlgrenska University Hospital, Östra Sjukhuset, Gothenburg, Sweden

**Keywords:** Exercise, Team sport, Ageing, Walking football, Injury incidence, Health

## Abstract

**Background:**

Walking football is a recreational sport that is aimed mainly at older adults and has been shown to be fun and social. However, reports from health care providers indicate that there is a lack of knowledge regarding the safety aspects of team sports for older adults, and the current research on injuries and well-being within walking football is limited. Thus, the aim of this study was to estimate the incidence and characteristics of injury among older adults with low physical activity levels starting to play walking football. A secondary aim was to descriptively characterise the participants’ self-rated physical and mental health.

**Methods:**

Thirteen men and twenty-six women (mean age of 74.0 ± 5.7 years) were enrolled. During 12 weeks of walking football, the participants completed a weekly injury questionnaire. At both baseline and after completing 12 weeks of walking football, the participants answered four questions assessing their self-rated physical health, mental health, anxiety, and loneliness. Thirty-three participants (11 men, 22 women) completed the study.

**Results:**

The participants’ ability to fully participate in playing walking football during the 12-week study period was 95% on average. Three out of 33 participants suffered from an injury leading to reduced or missing training sessions, an injury incidence proportion of 9%. The estimated injury incidence rate was 7.25 injuries per 1,000 player-hours (95% CI: 1.49–21.18). Mild or moderate pain was reported by 11 participants during the 12-week period. A total of 87.9% of the participants rated their general physical health as ‘good’ to ‘excellent’ at baseline, compared with 81.8% post-intervention, whereas 90.9% of the participants rated their general mental health as ‘good’ to ‘excellent’ compared with 93.9% post-intervention.

**Conclusions:**

This study presents preliminary descriptive estimates of injury incidence and self-rated physical and mental health during a 12-week walking football intervention. The findings should be interpreted with caution due to the small sample size and low number of injury events and should be confirmed in larger cohorts with longer follow-up periods.

**Trial registration:**

ISRCTN41037595, retrospectively registered 14/08/2024.

**Supplementary Information:**

The online version contains supplementary material available at https://doi.org/10.1186/s13102-026-02224-4.

## Introduction

The World Health Organization recommends that older adults aged 60 years or older perform 150–300 min of moderate-intensity aerobic activity per week to prevent and treat several common health-related diseases affecting both physical and mental health [1, 2]. However, in the European Union (EU), only 44.5% of individuals aged 65–74 years spend at least 180 min per week on physical activity, and this number decreases as people become older, falling to 33.4% for individuals aged 75 years or more [3]. A best-practice clinical model, titled *Physical activity on prescription (PAP)*, has been developed for individuals who need to increase their physical activity to prevent or treat illness. Physical activity is prescribed to insufficiently active patients in the healthcare setting, according to evidence presented in the Physical Activity in the Prevention and Treatment of Disease-book (FYSS in Swedish) [4], and the model has been spread to other European countries in a recent EU project [5]. The prescription is customized according to the disease as well as individual factors and barriers to activity and patient motivation. The actual intensity of a prescribed activity for a given condition thus depends on individual fitness. For older adults with lower fitness, being prescribed moderate-intensity activity can, for example, include Nordic walking. A lack of adherence is a major clinical problem when attempting behavioural change among patients [6]. Thus, there is also a need for activities where adherence is maintained.

Previous research has shown that team sports increase social interaction and togetherness [7, 8], which provides great social benefits for older adults. This could in turn increase the motivation of older adults and therefore also increase adherence with a given prescription of activity. However, certain team sports may not be optimal for physically untrained older adults because of safety aspects (e.g., injuries, medical complications), even if the intensity is suitable for treating the underlying condition. The Swedish Public Health Agency reported that health care providers lack knowledge regarding the safety aspects of team sports for older adults [9]. Walking football is a recreational sport that is aimed mainly at older adults and/or individuals who are not able to participate in regular football. Compared with regular football, it is played on a smaller pitch with no tackling allowed, played with or without goalkeepers, and with fewer players per team. Previous studies have shown that walking football is mainly a moderate- to vigorous-intensity aerobic activity [10–12]. It has also been shown that active walking football players generally have a better health profile than their age-equivalent peers do [10]. However, the current research on injuries and well-being within walking football is limited. Much of the previous research has focused on male individuals, non-beginners, middle-aged individuals, specific patient groups aimed at treating a specific disease, and/or already physically active individuals [10, 11, 13–19].

Thus, the overarching aim of this study was to estimate the incidence and characteristics of injury among older adults with low physical activity levels during a 12-week walking football intervention. A secondary aim was to descriptively characterise participants’ self-rated physical and mental health during the 12-week intervention. In addition, any medical adverse events during the intervention were recorded.

## Materials and methods

### Study design

This prospective descriptive study was conducted alongside a 12-week walking football intervention from August until the end of November 2023 following the TIDieR checklist for intervention studies (see supplementary file) [20]. The participants played walking football twice a week for 12 weeks in either central or southern Sweden. The present study describes prospectively collected data on participation, pain, injuries and medical adverse events during the 12-week period, as well as self-rated physical and mental health at baseline and post-intervention.

Two established football clubs in southern Sweden and two in central Sweden were contacted by the study leader, who agreed to start new walking football sections at each club. Details were announced on their respective websites and social media channels of upcoming information meetings about the study. Interested individuals could sign up for the information meeting by contacting the study leader. Three separate information meetings were held where the project was described, and the participants could ask questions.

The inclusion criterion was that the participants had to self-rate their everyday physical activity level as one or two on one to four grades of the Saltin-Grimby scale, representing having a sedentary lifestyle (one) or some physical activity for least 4 h per week (e.g., walking or gardening) (two) [21]. Furthermore, the participants were not allowed to have any previous experience playing walking football. The exclusion criterion was that the participants could not walk unrestrictedly (e.g., had to use a walker). Additional health outcomes assessed in the same cohort during the 12-week period have been previously reported [22]. Prior to enrolment, all participants signed an informed consent form.

### Self-rated health

At baseline, participants answered four self-reported questions regarding physical health, mental health, anxiety, and loneliness based on a previous study (see supplementary material). These questions have previously been used in The Swedish CArdioPulmonary bioImage Study (SCAPIS) [23] and in the current context of walking football [10].

The questions used five-point Likert scales (coded 1–5), and the participants had alternatives that ranged from, e.g., ‘excellent’ to ‘poor’ or ‘never’ to ‘very often’ depending on the question. The same four questions were administered after the 12-week period.

### Intervention

Walking football was played twice a week for 2 × 20 min for a total of 80 min per week over 12 consecutive weeks. In total, 24 sessions took place, and no sessions were cancelled because of adverse weather or low participant attendance. The participants conducted warm-up and cool-down for each session themselves with no standardised protocol used. The games were played on a pitch according to standardised measurements for walking football (15 to 20 m wide and 30 to 40 m long) and were adjusted according to the number of participants, if needed (from 4 v. 4 to 8 v. 8). During the sessions, no substitutes were used to ensure that all participants acquired the same dose of walking football in terms of minutes played during each session. All the sessions followed the Swedish Football Association’s rules and guidelines for walking football [24], meaning that no tackling and no goalkeepers were allowed. The players rotated between positions, with different teammates and mixed teams (men and women) in each session. All the sessions were refereed by the participants themselves, and all the sessions during the study period were played on artificial turf with a size 5 ball. A majority of the walking football sessions were attended by a test leader.

### Injury registration

All participants were given a booklet that consisted of 12 identical pages (one page for each week of the study period) with questions from the Oslo Sports Trauma Research Center Overuse Injury Questionnaire (OSTRC-O2) [25]. The OSTRC-O2 has been previously used for recording injuries in football. Further injury definition was based on the International Olympic Committee (IOC) consensus statement regarding methods for recording and reporting epidemiological data on injury and illness in sports [26] and its updated football-specific extension [27].

Injuries were recorded prospectively using the updated OSTRC-O2 questionnaire [25], with the Swedish wording based on the previously translated and validated Swedish version of the OSTRC questionnaire [28]. All the participants answered the questionnaire weekly during the 12-week intervention of playing walking football. As walking football is predominantly a recreational sport not aimed at competing it was decided within the research group to exclude question three from the OSTRC-O2 questionnaire regarding performance, as this was deemed difficult for the participants to answer. In the case of an injury that needed treatment, the participants first sought treatment at their local health centre. If the injury was deemed to need further treatment the medical doctor attached to the study could refer the participants to the local hospital.

Any physical complaint was only recorded as an injury if it resulted directly from participation in playing walking football, as noted by the participants.

### Participation, injury incidence proportion, and estimated injury incidence rate

The participants filled out the OSTRC-O2 questionnaire weekly if they had been able to participate in playing walking football in full, with some problems, reduced participation, or if they were unable to participate. For comparability with previous and future studies, the injury incidence proportion (cumulative incidence) and estimated injury incidence rate (person-time incidence rate) were also calculated. To calculate the injury incidence proportion for the 12-week period, the injured athletes were divided by the number of participating athletes. The estimated injury incidence rate per 1,000 player-hours of play was calculated by dividing the total number of recorded injuries by the total number of player-hours (exposure) and then multiplying by 1,000 [29]. In the OSTRC-O2 questionnaire, the participants filled out if they had to change their participation in the walking football sessions as part of the study. The options consisted of no change, to a lesser extent, to a moderate extent, to a great extent, or that they were not able to participate.

### Pain and injury severity

The severity of the injury was quantified as time-loss from full walking football participation. All injuries were classified into five categories (two categories were removed because they exceeded the study length of 12 weeks/84 days) to record how many days the participants were affected by the injury: 0 days, 1–3 days, 4–7 days, 8–28 days and 29–90 days. The quantification of time-loss stopped only when the injured participant returned to full unrestricted (no reduction) walking football play without modifications, as noted from the participants’ OSTRC-O2 questionnaire.

### Injury location

All injuries were classified into one of the fifteen different anatomical locations described in the football-specific extension of the IOC consensus statement [27].

### Medical adverse events

To assess the safety aspects of walking football, in addition to recording injuries, we recorded any serious medical adverse events (i.e., cardiovascular or other) during the 12-week intervention, including symptoms (i.e., chest pain, syncope and palpitations) reported by the participants.

### Ethical considerations

The study was conducted according to the WMA Declaration of Helsinki and was approved by the Swedish Ethical Review Authority (ref. numbers 2023-02309-01 and 2023-06704-02).

### Statistical analyses

The injury incidence proportion, incidence rate, injury severity, and injury location data, as well as data from the health questionnaire and OSTRC-O2 questionnaire, were analysed via descriptive statistics and are presented as percentage values and numerical values. The 95% CI was calculated using the exact Poisson (Garwood) method. All the data were collected in Microsoft Excel version 2302 (Microsoft Corporation, Redmond, WA, USA) and subsequently transferred to SPSS for additional analysis (SPSS Version 29, IBM Inc., Armonk, New York).

## Results

A total of 39 participants (men, *n* = 13; women, *n* = 26; mean age of 74.0 ± 5.7, range 64–85 years) were enrolled in the study. Of these, 33 completed the post-intervention and the OSTRC-O2 questionnaire. Six participants did not complete the post-intervention for personal reasons (*n* = 5) or because of longer illness (*n* = 1). Characteristics of the 33 participants included in the present analyses are presented in Table 1.

**Table 1:** Characteristics of the study population

	All (*n* = 33)	Men (*n* = 11)	Women (*n* = 22)
Age (year)	74.0 ± 5.7 (64–85)	75.0 ± 6.3 (65–84)	73.4 ± 5.4 (64–85)
Height (cm)	169 ± 8.3 (158–190)	176 ± 9.0 (164–190)	165 ± 4.6 (158–174)
Body mass (kg)	74.0 ± 15.6 (50.5–105)	79.3 ± 12.5 (63.4–104.1)	71.4 ± 16.6 (50.5–105)
BMI (kg∙m^− 2^)	26.0 ± 4.7 (19.4–36.5)	25.5 ± 3.2 (21.8–30.5)	26.2 ± 5.3 (19.4–36.5)
Waist circumference (cm)	93.9 ± 13.9 (67–123)	97.8 ± 10.9 (81–114)	91.9 ± 15.0 (67–123)
Use of prescribed drugs	79% (*n* = 26)	82% (*n* = 9)	77% (*n* = 17)
Known hypertension (self-reported)	42% (*n* = 14)	55% (*n* = 6)	36% (*n* = 8)

Among the 33 participants, 64% lived by themselves, 36% lived together with a partner or friend, and all of them were old-age pensioners. The educational level differed among the participants, as 42% had a degree from a university or college, 46% had finished upper secondary school, and 12% had finished elementary school. None of the participants were smokers; however, 36% had smoked previously in their life but had now quit.

### Participation, injury incidence proportion, and estimated injury incidence rate

The ability to fully participate during the 12 weeks of walking football was 95% on average (Table 2). Out of 24 possible sessions, the number of walking football sessions attended by the participants during the intervention was 18.4 ± 4.4 [22]. In total, the participants played 621.5 out of a possible 792 sessions, leading to 414 exposure hours for the cohort [29].

**Table 2:** Data from OSTRC-O2 questionnaire (*n* = 33) W = Week

Participation in walking football	W1	W2	W3	W4	W5	W6	W7	W8	W9	W10	W11	W12
Full participation	97% (*n* = 32)	97% (*n* = 32)	91% (*n* = 30)	94% (*n* = 31)	94% (*n* = 31)	94% (*n* = 31)	97% (*n* = 32)	97% (*n* = 32)	94% (*n* = 31)	94% (*n* = 31)	97% (*n* = 32)	100% (*n* = 33)
Full participation but some problems	0% (*n* = 0)	0% (*n* = 0)	3% (*n* = 1)	3% (*n* = 1)	3% (*n* = 1)	3% (*n* = 1)	3% (*n* = 1)	0% (*n* = 0)	0% (*n* = 0)	0% (*n* = 0)	3% (*n* = 1)	0% (*n* = 0)
Reduced participation	3% (*n* = 1)	3% (*n* = 1)	6% (*n* = 2)	3% (*n* = 1)	0% (*n* = 0)	3% (*n* = 1)	0% (*n* = 0)	3% (*n* = 1)	0% (*n* = 0)	3% (*n* = 1)	0% (*n* = 0)	0% (*n* = 0)
No participation	0% (*n* = 0)	0% (*n* = 0)	0% (*n* = 0)	0% (*n* = 0)	3% (*n* = 1)	0% (*n* = 0)	0% (*n* = 0)	0% (*n* = 0)	6% (*n* = 2)	3% (*n* = 1)	0% (*n* = 0)	0% (*n* = 0)
**Change of regular training of walking football**												
No change	97% (*n* = 32)	97% (*n* = 32)	94% (*n* = 31)	97% (*n* = 32)	94% (*n* = 31)	97% (*n* = 32)	97% (*n* = 32)	97% (*n* = 32)	94% (*n* = 31)	94% (*n* = 31)	100% (*n* = 33)	100% (*n* = 33)
To a lesser extent	0% (*n* = 0)	0% (*n* = 0)	0% (*n* = 0)	3% (*n* = 1)	3% (*n* = 1)	0% (*n* = 0)	0% (*n* = 0)	0% (*n* = 0)	0% (*n* = 0)	3% (*n* = 1)	0% (*n* = 0)	0% (*n* = 0)
To a moderate extent	0% (*n* = 0)	0% (*n* = 0)	0% (*n* = 0)	0% (*n* = 0)	0% (*n* = 0)	3% (*n* = 1)	3% (*n* = 1)	3% (*n* = 1)	0% (*n* = 0)	0% (*n* = 0)	0% (*n* = 0)	0% (*n* = 0)
To a great extent	0% (*n* = 0)	0% (*n* = 0)	0% (*n* = 0)	0% (*n* = 0)	0% (*n* = 0)	0% (*n* = 0)	0% (*n* = 0)	0% (*n* = 0)	0% (*n* = 0)	0% (*n* = 0)	0% (*n* = 0)	0% (*n* = 0)
I could not participate at all	3% (*n* = 1)	3% (*n* = 1)	6% (*n* = 2)	0% (*n* = 0)	3% (*n* = 1)	0% (*n* = 0)	0% (*n* = 0)	0% (*n* = 0)	6% (*n* = 2)	3% (*n* = 1)	0% (*n* = 0)	0% (*n* = 0)
**​Pain due to walking football​**​​												
No pain	88% (*n* = 29)	91% (*n* = 30)	88% (*n* = 29)	82% (*n* = 27)	91% (*n* = 30)	91% (*n* = 30)	94% (*n* = 31)	94% (*n* = 31)	94% (*n* = 31)	94% (*n* = 31)	94% (*n* = 31)	97% (*n* = 32)
Mild pain	6% (*n* = 2)	6% (*n* = 2)	6% (*n* = 2)	12% (*n* = 4)	3% (*n* = 1)	6% (*n* = 2)	3% (*n* = 1)	3% (*n* = 1)	3% (*n* = 1)	3% (*n* = 1)	6% (*n* = 2)	3% (*n* = 1)
Moderate pain	6% (*n* = 2)	3% (*n* = 1)	6% (*n* = 2)	6% (*n* = 2)	6% (*n* = 2)	3% (*n* = 1)	3% (*n* = 1)	3% (*n* = 1)	3% (*n* = 1)	3% (*n* = 1)	0% (*n* = 0)	0% (*n* = 0)
Severe pain	0% (*n* = 0)	0% (*n* = 0)	0% (*n* = 0)	0% (*n* = 0)	0% (*n* = 0)	0% (*n* = 0)	0% (*n* = 0)	0% (*n* = 0)	0% (*n* = 0)	0% (*n* = 0)	0% (*n* = 0)	0% (*n* = 0)

Only during weeks 5, 9 and 10 were there participants who were unable to participate in any of the two scheduled sessions due to injury. Three out of 33 participants sustained an injury resulting in reduced or missed participation in walking football, which corresponds to an injury incidence proportion of 9% for the population. The estimated injury incidence rate was 7.25 injuries per 1,000 player-hours (95% CI: 1.49–21.18).

During the 12-week intervention, three participants changed their regular training in walking football, ranging from a change of lesser extent to no participation at all. The change in regular training could, for example, include a reduction in play time to one half instead of two, as noted in their OSTRC-O2 questionnaire.

### Pain and injury severity

No participants reported severe pain during the 12-week intervention; however, 11 participants reported mild pain or moderate pain. All four participants reporting moderate pain during the 12-week intervention also reported mild pain during at least one week. Mild and moderate pain was reported most often at the beginning of the intervention but decreased from week 5 to the end of the intervention (Table 2). For the three recorded injuries, the severity of injury was 8–28 days (*n* = 2) and 29–90 days (*n* = 1). For the latter, the participant developed knee pain during the study period, allowing the participant to play only once a week instead of the scheduled two sessions per week (Table 2).

### Injury location

The injury locations among the participants were the knee (*n* = 2) and hip (*n* = 1). Other complaints that were noted but did not lead to a reduction in walking football participation were the thigh (*n* = 2), hip (*n* = 1) and toe (*n* = 1).

### Medical adverse events

No medical adverse events or significant symptoms were reported during the study.

### Self-rated health

Participants’ self-rated physical and mental health at baseline and post-intervention are presented below in Figs​. 1, 2, 3 and 4.

**Fig. 1:**
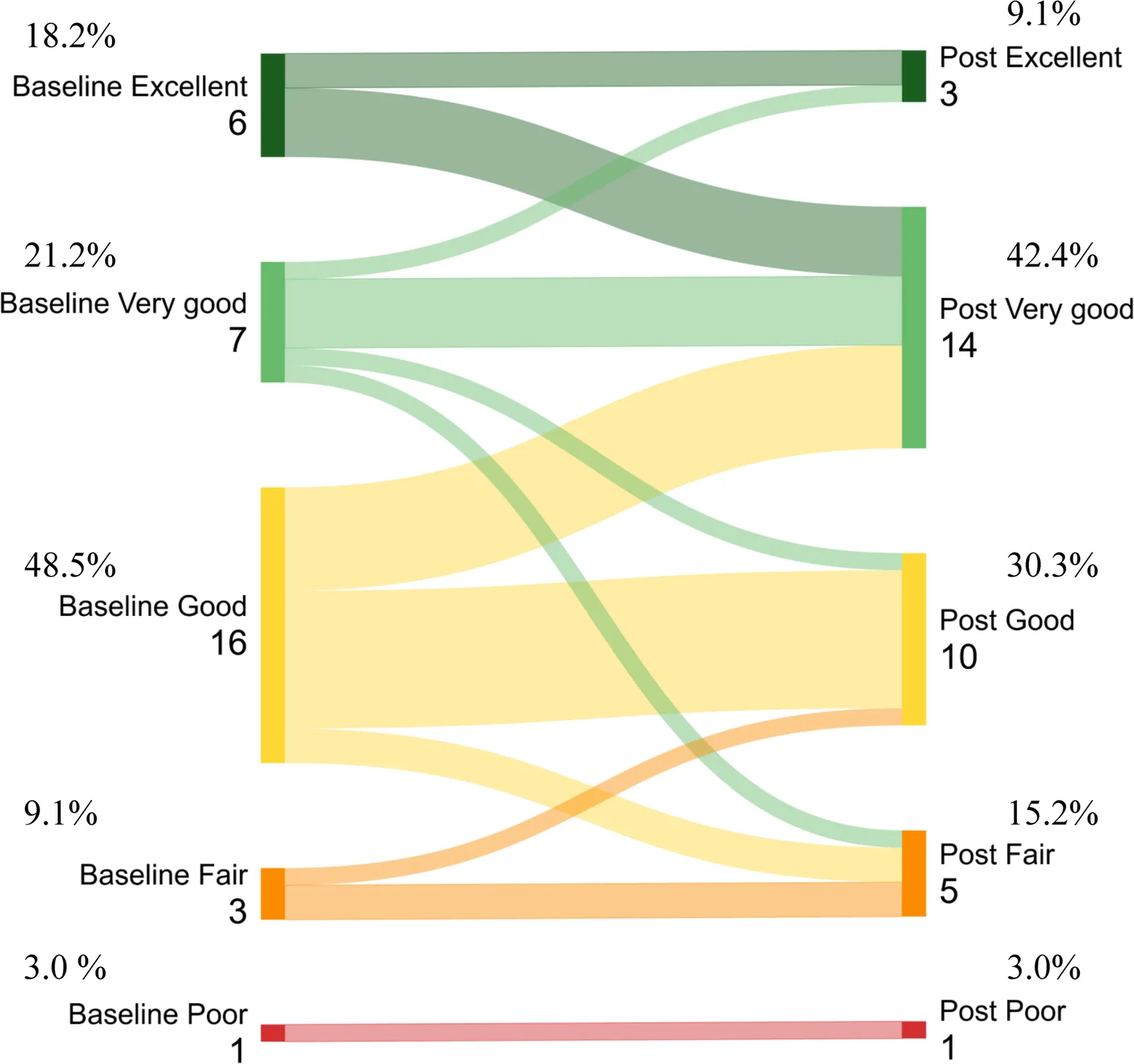
Participants’ self-rated physical health (*n*=33)

Eighty-seven-point nine percent of the participants rated their general physical health as ‘good’ to ‘excellent’ at baseline, compared with 81.8% post-intervention (Fig. 1).

**Fig. 2:**
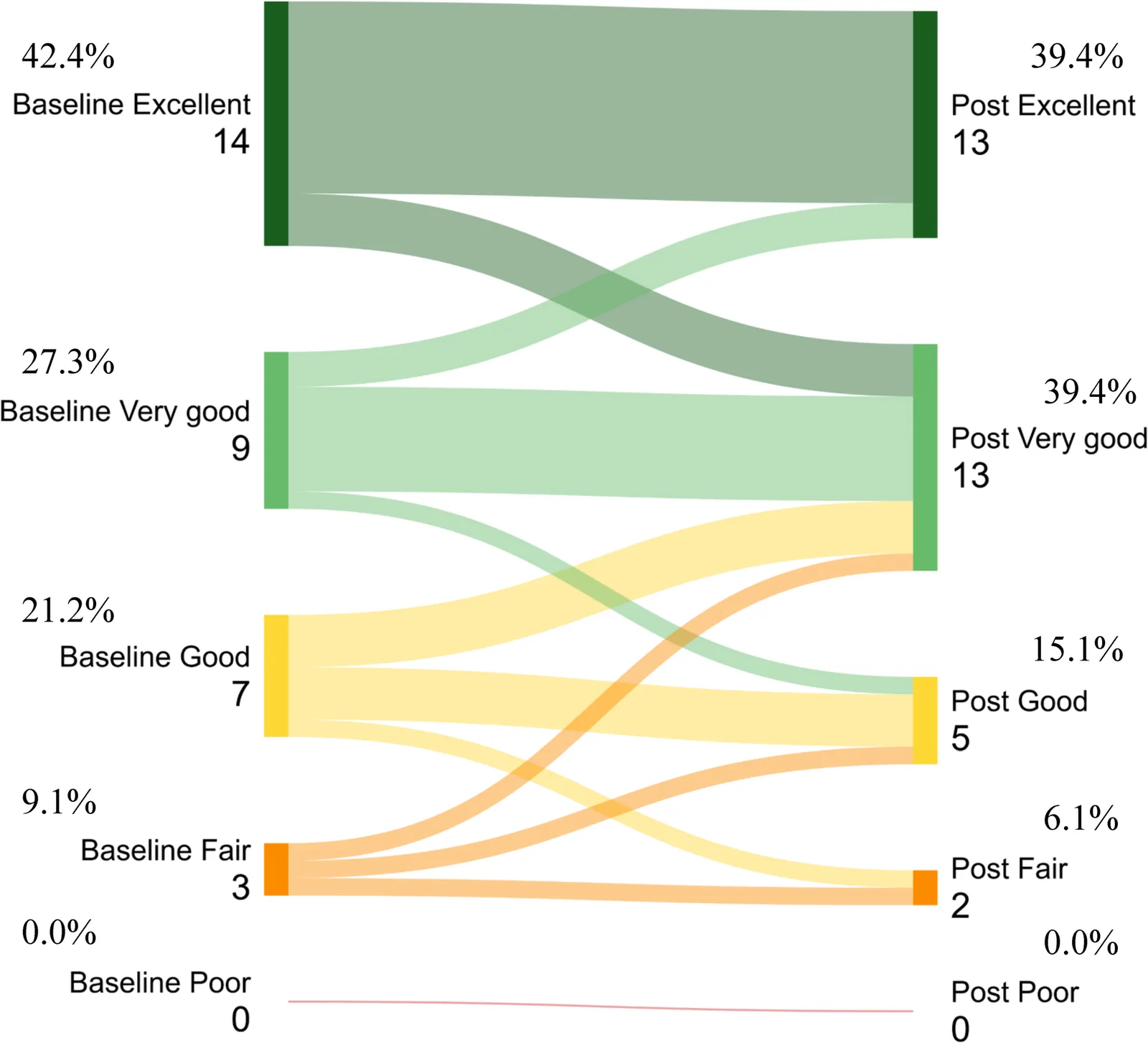
Participants’ self-rated mental health (*n*=33)

Ninety-point nine percent rated their general mental health as ‘good’ to ‘excellent’, compared with 93.9% post-intervention (Fig. 2).

**Fig. 3:**
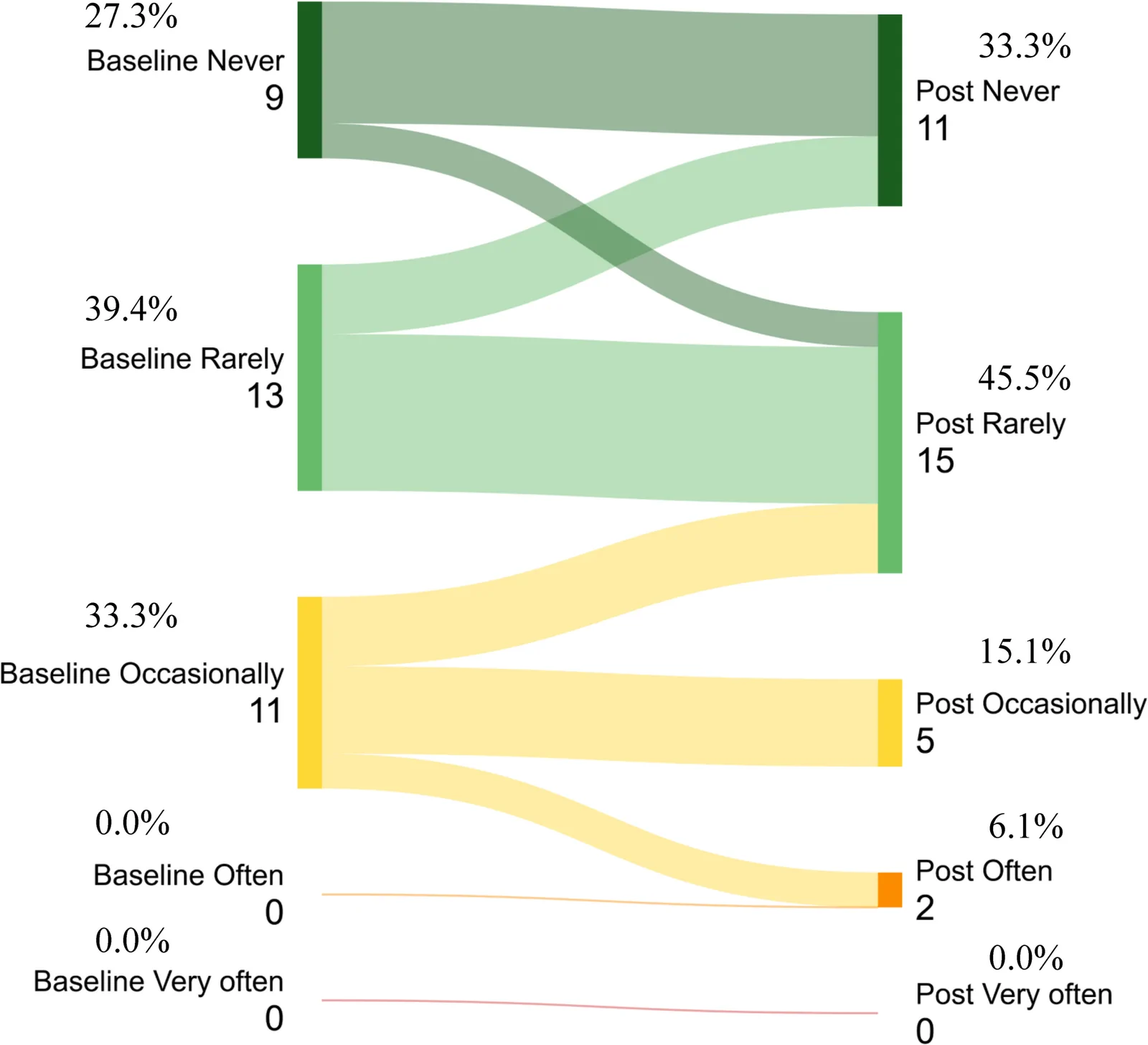
Participants’ self-rated feelings of anxiety and worry (*n*=33)

The participants’ experience of anxiety or worry at baseline was relatively low, as 66.7% selected the option ‘never’ or ‘rarely’, compared with 78.8% post-intervention (Fig. 3).

**Fig. 4:**
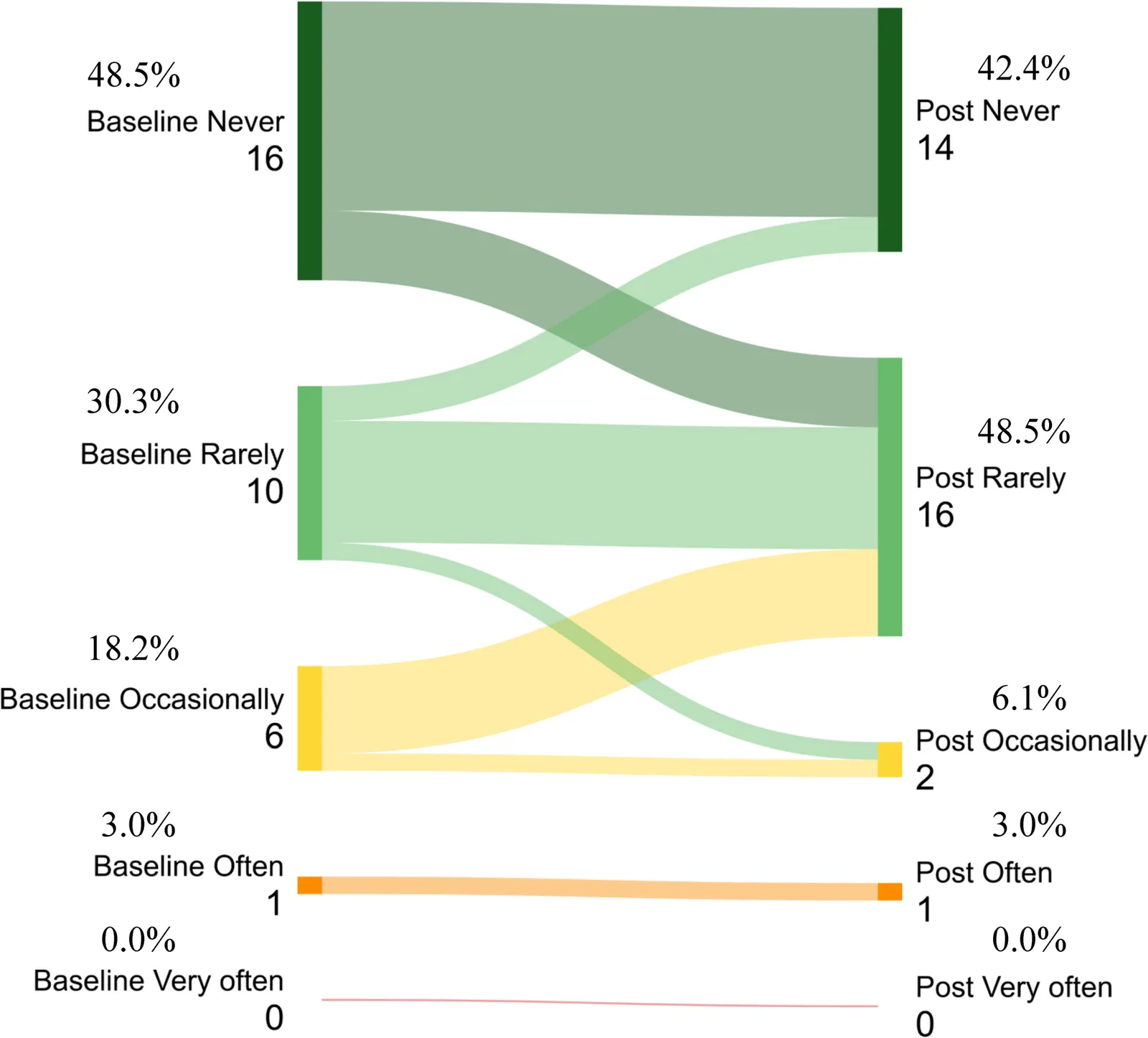
Participants’ self-rated feelings of being alone (*n*=33)

At baseline, 78.8% of the participants rated their feelings of loneliness ‘never’ or ‘rarely’ compared with 90.9% post-intervention (Fig. 4).

## Discussion

The aim of this study was to estimate the incidence and characteristics of injury among older adults with low physical activity levels during a 12-week walking football intervention. A secondary aim was to descriptively characterise participants’ self-rated physical and mental health during the 12-week intervention. The main finding of the present study was the low number of injuries sustained by the participants during the 12-week intervention. Small differences in the participants’ self-rated physical or mental health parameters were observed. In addition, no serious symptoms or medical adverse events were reported during the intervention.

Pain was more prevalent at the beginning of the intervention but decreased over time. However, as the present study was descriptive and did not include a control group it is not possible to determine the cause of this pattern.

The results regarding injuries from the current study are comparable to the results of Reddy et al. (2017), who reported that three participants missed walking football sessions because of injuries in the same time period (12 weeks). It was also common for the participants to initially experience pain and stiffness which gradually subsided. However, the mean age of the participants was lower (mean age 61.1 years compared with 74.0 years in the present study), which means that they could have been more resistant to injuries, as both muscle mass and aerobic capacity decreases (up to 20% per decade) with increasing age [30]. A recent study observed an overall injury incidence of 49.4 injuries per 1,000 h during the FA walking football cup [31], while a four-month observational study in seven walking football clubs in the UK observed an injury incidence of 5.3 (CI: 1.5–11.5) per 1,000 h in training and 37.6 (CI: 8.7–83.9) per 1,000 h in matches [32]. In comparison, a study on recreational football reported an injury incidence of 7.8 injuries per 1,000 h of recreational football participation (both training and match play) for individuals aged 15–74 years [33].

Differences in reported injury incidences may be influenced by characteristics of the study populations, such as if the players were beginners or experienced players, age (ranging from 40 + to 70+), as well as whether injuries were recorded during training or match play. Furthermore, the small number of injuries in the present study and the wide confidence interval limit the interpretation of comparisons with previous studies. These comparisons should be interpreted as descriptive and not indicative that walking football is a safe sport.

Small differences in the participants’ self-rated physical and mental health were observed between baseline and the end of the intervention period of 12 weeks.

The proportion of participants rating their physical health as very good or excellent at baseline was 39.4%, compared with 51.5% at post-intervention; this proportion is comparable to that of active walking football players, who rated their physical health as very good or excellent at 47.0% [10]. Direct comparisons between the studies should be interpreted with caution due to the differences in study populations and the descriptive nature of the present study.

### Clinical implications

The present findings provide preliminary information on injury incidence and participation in walking football among older adults with low physical activity levels. Although a very limited number of injuries were recorded and no medical adverse events occurred, the small sample size and limited follow-up period prevent firm conclusions regarding the safety of walking football. Larger prospective studies are needed before recommendations regarding its suitability as prescribed physical activity can be made based on injury risk.

### Limitations and strengths

The limitations of the study include the limited sample of participants and the limited number of recorded injuries which resulted in a wide confidence interval around the estimated injury incidence. Furthermore, the self-reporting of injuries may be subject to reporting bias and under-reporting of minor injuries or health complaints. The absence of a control group and lack of inferential analyses of self-rated health outcomes prevent conclusions regarding any effects of walking football on physical and mental health. Furthermore, the relatively short follow-up period limits the ability to assess longer-term injury occurrence and health outcomes. The relatively high self-rated physical and mental health at baseline should be considered when interpreting the differences between baseline and post-intervention. Owing to the exclusion of question three from the OSTRC-O2 questionnaire, it was not possible to calculate a severity score for the participants.

A strength of the present study is the inclusion of older adults (60+) with low physical activity levels, both men and women, with no prior experience of regular walking football play. Furthermore, injury data were recorded using standardised methods based on the football-specific extension of the IOC consensus statement.

## Conclusion

This study presents preliminary descriptive estimates of injury incidence and self-rated physical and mental health during a 12-week walking football intervention. Three injuries resulting in reduced participation were recorded, corresponding to an estimated injury incidence rate of 7.25 injuries per 1,000 player-hours (95% CI: 1.49–21.18). Participants generally reported favourable perceptions of their physical and mental health throughout the study period. Future studies should implement a randomised controlled trial design with a larger cohort over a longer follow-up period.

## Supplementary Information

Supplementary Material 1.

## Data Availability

The data that support the findings of this study is available from the corresponding author, AC, upon reasonable request.

## Abbreviations

EU: European Union
FYSS: Physical Activity in the Prevention and Treatment of Disease
IOC: International Olympic Committee
OSTRC-O2: Oslo Sports Trauma Research Center Overuse Injury Questionnaire
PAP: Physical activity on prescription
SCAPIS: The Swedish CArdioPulmonary bioImage Study
